# Automatic contour propagation using deformable image registration to determine delivered dose to spinal cord in head-and-neck cancer radiotherapy

**DOI:** 10.1088/1361-6560/aa76aa

**Published:** 2017-07-12

**Authors:** P L Yeap, D J Noble, K Harrison, A M Bates, N G Burnet, R Jena, M Romanchikova, M P F Sutcliffe, S J Thomas, G C Barnett, R J Benson, S J Jefferies, M A Parker

**Affiliations:** 1 Cavendish Laboratory, University of Cambridge, JJ Thomson Avenue, Cambridge, CB3 0HE, United Kingdom; 2 Department of Oncology, University of Cambridge, Hills Road, Cambridge, CB2 0QQ, United Kingdom; 3 Department of Oncology, Addenbrooke’s Hospital, Hills Road, Cambridge, CB2 0QQ, United Kingdom; 4 Department of Medical Physics and Clinical Engineering, Addenbrooke’s Hospital, Hills Road, Cambridge, CB2 0QQ, United Kingdom; 5 Department of Engineering, University of Cambridge, Trumpington Street, Cambridge, CB2 1PZ, United Kingdom; ply21@hep.phy.cam.ac.uk

**Keywords:** deformable image registration, contour propagation, head-and-neck cancers, spinal cord, delivered dose, toxicity, adaptive radiotherapy

## Abstract

To determine delivered dose to the spinal cord, a technique has been developed to propagate manual contours from kilovoltage computed-tomography (kVCT) scans for treatment planning to megavoltage computed-tomography (MVCT) guidance scans. The technique uses the Elastix software to perform intensity-based deformable image registration of each kVCT scan to the associated MVCT scans. The registration transform is then applied to contours of the spinal cord drawn manually on the kVCT scan, to obtain contour positions on the MVCT scans. Different registration strategies have been investigated, with performance evaluated by comparing the resulting auto-contours with manual contours, drawn by oncologists. The comparison metrics include the conformity index (CI), and the distance between centres (DBC). With optimised registration, auto-contours generally agree well with manual contours. Considering all 30 MVCT scans for each of three patients, the median CI is $0.759 \pm 0.003$, and the median DBC is ($0.87 \pm 0.01$) mm. An intra-observer comparison for the same scans gives a median CI of $0.820 \pm 0.002$ and a DBC of ($0.64 \pm 0.01$) mm. Good levels of conformity are also obtained when auto-contours are compared with manual contours from one observer for a single MVCT scan for each of 30 patients, and when they are compared with manual contours from six observers for two MVCT scans for each of three patients. Using the auto-contours to estimate organ position at treatment time, a preliminary study of 33 patients who underwent radiotherapy for head-and-neck cancers indicates good agreement between planned and delivered dose to the spinal cord.

## 1. Introduction

Patients undergoing radical radiotherapy for head-and-neck cancers (HNC) frequently experience weight loss during the course of treatment (Ottosson *et al* 2013). In addition, many structures within the treated volume, including primary and nodal disease, and organs at risk, may undergo substantial changes in position, shape and size during this period (Marzi *et al* 2012). Cumulative radiation doses to these structures can then differ from those expected from the initial planning process (Loo *et al* 2011). Understanding these differences, and developing strategies to manage the problems that can ensue, are objectives of adaptive radiotherapy, which is the subject of much current research (Gregoire *et al* 2015).

With increasing survival rates for HNC patients (Pulte and Brenner 2010), a key objective is to reduce long-term side effects (toxicity) from radiotherapy. The VoxTox research programme aims to investigate differences between planned and delivered dose to millimetre-scale volume elements (voxels) of the organs at risk, and to correlate delivered dose with toxicity. The spinal cord is often the dose-limiting structure in HNC. Exceeding the tolerance dose increases the risk of Lhermitte’s syndrome, and of catastrophic sequelae, such as transverse myelitis (Kirkpatrick *et al* 2010, Pak *et al* 2012). Changes in patient anatomy over a course of treatment may lead to differences between planned dose and delivered dose (Duma *et al* 2012). If the delivered dose to the cord is higher than planned, care must be taken to ensure that the dose does not exceed tolerances. If the delivered dose is lower than planned, there is potential for dose escalation to the tumour.

HNC patients typically receive from 30 to 35 radiotherapy fractions, so that manually contouring the spinal cord on all computed-tomography (CT) scans recorded at treatment time for image guidance would be a laborious task. The image-guidance scans also exhibit poor signal-to-noise ratios, and manual contours are subject to inter-observer variability (Song *et al* 2006). There is, then, a need for consistent and accurate automated contouring. The technique described in this work has been developed to locate the spinal cord on megavoltage computed-tomography (MVCT) guidance scans by propagating manual contours on kilovoltage computed-tomography (kVCT) planning scans. The contour propagation relies on intensity-based deformable image registration, performed using the Elastix (Klein *et al* 2010) software. Similar approaches have been used to propagate manual contours for tumours and for parotid glands (Al-Mayah *et al* 2010, Faggiano *et al* 2011). To our knowledge, there are no reports of this approach being applied to the spinal cord, although some commercial systems offer atlas-based segmentation of the spinal canal.

## 2. Materials and methods

### 2.1. Patient data

The VoxTox study has ethical approval from the National Research Ethics Committee East of England (13/EE/0008). The present analysis relates to data from 33 consented patients, treated at Addenbrooke’s Hospital, Cambridge, UK, using the Hi-Art system (TomoTherapy, Madison, WI). These patients underwent radical radiotherapy for primary squamous cell carcinomas of the pharynx and larynx, with a prescribed dose of 65 Gy in 30 fractions or 68 Gy in 34 fractions, with or without concurrent chemotherapy. All patients receiving radical radiotherapy for HNC at our centre undergo daily MVCT image-guidance.

Each voxel in the original kVCT scans has a dimension of ${1.074}~{\rm mm} \times {1.074}~{\rm mm}~\times$${3.000}~{\rm mm}$, and is down-sampled to ${2.148}~{\rm mm} \times {2.148}~{\rm mm} \times {3.000}~{\rm mm}$ in the archival scans used in the present study. The voxel size of the MVCT scans is ${0.754}~{\rm mm}~ \times $$ {0.754}~{\rm mm} \times {6.000}~{\rm mm}$.

For all 33 patients considered, the spinal cord was manually contoured on the down-sampled kVCT scans by a single radiation oncologist, who has four years’ experience treating cancers of the head and neck. For an initial set of three patients, this oncologist manually outlined the spinal cord twice on each MVCT scan, at intervals of four to eight weeks, allowing evaluation both of our technique for automated contouring and of intra-observer variability. For the 30 other patients, the same oncologist manually outlined the spinal cord once on a randomly selected MVCT scan per patient, allowing validation of the automated contouring against a broader range of patient anatomies.

Five additional oncologists, experienced at treating tumours of the head-and-neck region, provided manual contours on two MVCT scans per patient for the initial set of three patients. This meant that the spinal cord was outlined on six MVCT scans by a total of six oncolgists, allowing investigation of inter-observer variability.

### 2.2. Contour propagation using deformable image registration

Deformable image registration has been performed using the Elastix (Klein *et al* 2010) software, which is largely based on the insight segmentation and registration toolkit (ITK) (Ibanez *et al* 2003). CT scans from Addenbrooke’s Hospital are stored as sets of image slices in the format of Digital Imaging and Communications in Medicine (DICOM). Since Elastix registration is performed on three-dimensional scans, the image slices are first combined by converting into the format of the Neuroimaging Informatics Technology Initiative (NIfTI). A mask is then created around the region occupied by the patient’s head and upper body on each kVCT scan, so as to exclude the couch from the registration process. The mask is constructed by setting a threshold intensity value that separates patient and couch from air, and by then selecting the largest group of contiguous above-threshold voxels.

Registration aligns one image, termed the moving image, with another image, termed the fixed image. As a function of spatial coordinate, ${\bf x}$, these images are taken to have grey-level intensities $I_{M}({\bf x})$ and $I_{F}({\bf x})$ respectively. The registration process then identifies the values of the parameters of a transformation, ${\bf T(x)}$, such that $I_{M}({\bf T(x)})$ is aligned with $I_{F}({\bf x})$, according to a suitable metric.

In the present work, MVCT scans have been registered to kVCT scans, and the grey-level intensities have been in Hounsfield units. Different types of registration transforms have been considered in this study, including translations, affine transforms, and B-spline transforms. The last mentioned has been optimised with respect to control-point spacing: too high a value can result in small structures being disregarded, but too low a value can result in erratic behaviour.

Registrations have been performed using an iterative approach, based on gradient-descent optimistation of a mutual-information (Mattes *et al* 2003) similarity metric. For each patient considered, the points defining the contour of the spinal cord on the kVCT scan are mapped to the MVCT scans, by using Transformix (Klein *et al* 2010), a component in Elastix, to apply the relevant registration transform. The resulting auto-contours are saved as structure sets, in DICOM format.

### 2.3. Conformity analysis

Comparisons have been made, slice by slice, between manually drawn contours and auto-contours, and between different sets of manually drawn contours. The metrics considered are the conformity index (CI), also known as the Jaccard similarity coefficient (Hanna *et al* 2010), the distance to conformity (DTC) (Jena *et al* 2010), the difference in left–right dimension, the difference in anterior-posterior dimension, and the distance between centres (DBC).

To study inter-observer variability, contours from each of the six observers are compared with the contours from the other five. For each conformity metric, the average of the resulting 15 values has been taken as the best estimate.

### 2.4. Calculation of delivered dose

The planned dose for a patient treated at Addenbrooke’s Hospital is calculated from the patient’s kVCT scan and treatment plan, using the proprietary software of the Hi-Art system. For the present study, we have calculated planned and delivered doses from kVCT and MVCT scans, using our own software, CheckTomo (Thomas *et al* 2016), which takes into account couch shifts between an MVCT scan and treatment delivery. Differences between doses from the Hi-Art system and doses from CheckTomo are small. For example, the mean difference between the two in the mean dose within the 50% isodose has been found to be $+1.1\%$ (range $-0.4\%$ to $+3.1\%$) (Thomas *et al* 2011).

The actual delivered dose, *D*_*A*_, for a voxel in the spinal cord on a patient’s kVCT scan has been obtained by summing over the fractionated doses to the corresponding voxel on each MVCT scan. The correspondence is determined using the relevant registration transform. Only the region covered by all of a patient’s MVCT scans is considered.

## 3. Results and discussions

### 3.1. Optimisation of registration parameters

**Figure 1.**
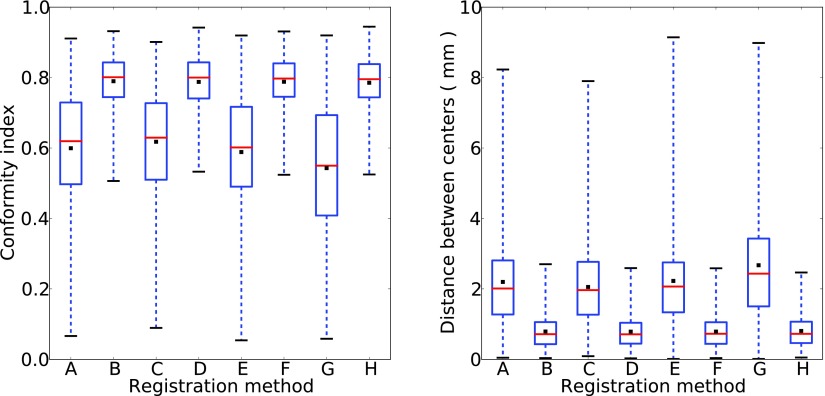
Box-and-whisker plots showing the effect, for a randomly selected patient, of different types of transformation on: (left) conformity index and (right) distance between centres. The transformations used are: (A) affine, (B) affine plus B-spline, (C) radiographer couch shifts, (D) radiographer couch shifts plus B-spline, (E) translations plus rotations, (F) translations plus rotations plus B-spline, (G) translation, (H) translation plus B-spline. Results for each transformation include: the mean (black square), the median (red line), first and third quartiles (respectively the bottom and the top of the blue box), and the lowest and highest values (respectively the bottom and top of the whiskers).

We first investigated the importance of non-rigid transformations, considering a randomly selected patient from the initial set of three. We compared the CI and DBC of auto-contours relative to manual contours, for auto-contours generated with translations, with translations plus rotations, with affine transforms, or with radiographer couch shifts. Each of these types of transformation has also been tried together with a non-rigid B-spline transform. The resulting box-and-whisker plots (figure 1) show that that the median CI of rigid-body and affine transformations by themselves is about 0.6, while the median DBC can be up to 2.6 mm. Additionally performing a B-spline transform increases the median CI to about 0.8, and reduces the median DBC to less than 1 mm. These results support use of B-spline transforms. Best results were obtained with an affine transform followed by a B-spline transform. In this case, the CI had median $0.801 \pm 0.004$ and mean $0.790 \pm 0.003$, the DBC had median ($0.71 \pm 0.02$) mm and mean ($0.79 \pm 0.02$) mm.

**Figure 2.**
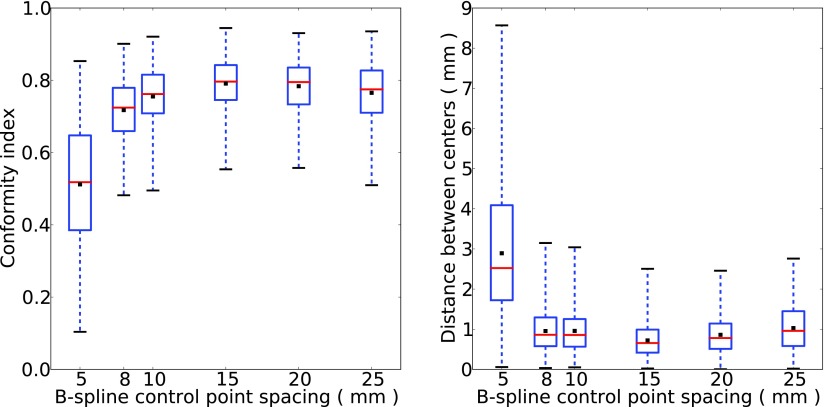
Box-and-whisker plots showing the effect, for a randomly selected patient, of varying the B-spline control-point spacing from 5 mm to 25 mm, on: (left) conformity index and (right) distance between centres.

All subsequent analysis has therefore been based on registration using an affine transform followed by a B-spline transform with optimised control-point spacing. The optimisation was achieved by varying the spacing from 5 mm to 25 mm. The box-and-whiskers plots (figure 2) for different spacing show that a value of 15 mm gives the highest mean CI and the smallest mean DBC.

**Figure 3.**
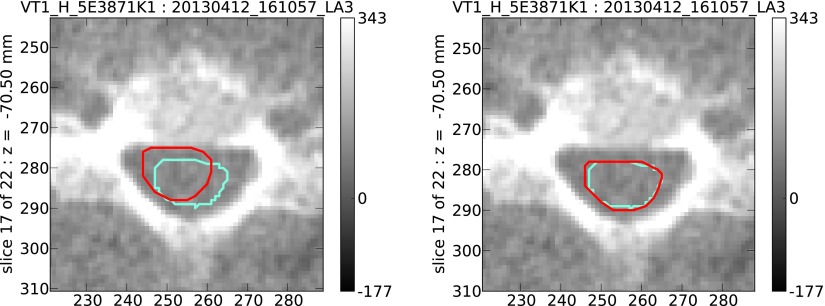
Spinal cord contours on a single MVCT scan slice; manual contours (cyan) and contours propagated form the patient’s kVCT scan (red). In this example, agreement between the manual contour and a propagated contour using an affine plus B-spline transform (right image, CI 0.88) is significantly better than agreement between the manual contour and the propagated contour using radiographer couch shifts (left image, CI 0.49).

An example is shown (figure 3) of a case where the auto-contouring significantly improves on the result obtained using radiographer couch shifts alone.

### 3.2. Conformity analysis: three patients

Auto-contouring has been performed for the set of three patients for whom the spinal cord has been manually outlined twice on each MVCT scan. This dataset consists of a total of 90 MVCT scans and 2107 slices. Comparing the auto-contours with one set of manual contours (table 1): the CI has median $0.759 \pm 0.003$ and mean $0.744 \pm 0.002$, the DBC has median ($0.87 \pm 0.01$) mm and mean ($0.95 \pm 0.01$) mm, the mean DTC is ($0.18 \pm 0.01$) mm, the mean difference in left–right dimensions is ($-0.38 \pm 0.03$) mm, and the mean difference in anterior-posterior dimensions is ($0.60 \pm 0.03$) mm. The mean differences can be compared with the pixel dimensions of an MVCT slice, of ${0.754}~{\rm mm} \times {0.754}~{\rm mm}$.

**Table 1.** Results from outlining the spinal cord on the MVCT scans of three patients. Each patient has 30 scans, giving 90 scans in total. Comparisons have been made between auto-contours and manually drawn contours (upper row for each parameter) and between two sets of manual contours from an intra-observer study (lower row for each parameter). The numbers of MVCT scans and slices relative to each comparison are shown. Results are given for the median, mean and standard deviation of the conformity index, and of the distance between centres (mm); and for the mean of the distance (mm) to conformity, of the difference (mm) in left–right dimensions, and of the difference (mm) in anterior–posterior dimensions. Quoted uncertainties are statistical.

Patient	1	2	3	Total
Slices	567	801	739	2107
	558	801	749	2108
Median CI	$0.801 \pm 0.004$	$0.762 \pm 0.004$	$0.719 \pm 0.005$	$0.759 \pm 0.003$
	$0.836 \pm 0.003$	$0.813 \pm 0.003$	$0.814 \pm 0.003$	$0.820 \pm 0.002$
Mean CI	$0.790 \pm 0.003$	$0.752 \pm 0.003$	$0.701 \pm 0.004$	$0.744 \pm 0.002$
	$0.825 \pm 0.003$	$0.805 \pm 0.002$	$0.804 \pm 0.003$	$0.810 \pm 0.002$
Std Dev CI	0.072	0.082	0.114	0.099
	0.059	0.068	0.076	0.070
Median DBC	$0.71 \pm 0.02$	$0.88 \pm 0.02$	$1.00 \pm 0.03$	$0.87 \pm 0.01$
	$0.63 \pm 0.02$	$0.66 \pm 0.02$	$0.60 \pm 0.02$	$0.64 \pm 0.01$
Mean DBC	$0.79 \pm 0.02$	$0.92 \pm 0.02$	$1.09 \pm 0.02$	$0.95 \pm 0.01$
	$0.69 \pm 0.02$	$0.71 \pm 0.01$	$0.68 \pm 0.01$	$0.69 \pm 0.01$
Std Dev DBC	0.47	0.47	0.62	0.54
	0.36	0.38	0.39	0.38
Mean DTC	$0.13 \pm 0.02$	$0.16 \pm 0.01$	$0.24 \pm 0.02$	$0.18 \pm 0.01$
	$0.02 \pm 0.01$	$0.04 \pm 0.01$	$0.01 \pm 0.01$	$0.03 \pm 0.01$
Mean L-R diff	$0.30 \pm 0.06$	$-0.44 \pm 0.04$	$-0.85 \pm 0.05$	$-0.38 \pm 0.03$
	$-0.28 \pm 0.04$	$-0.20 \pm 0.03$	$-0.28 \pm 0.04$	$-0.25 \pm 0.02$
Mean A-P diff	$0.13 \pm 0.04$	$0.62 \pm 0.04$	$0.93 \pm 0.06$	$0.60 \pm 0.03$
	$0.21 \pm 0.03$	$0.33 \pm 0.03$	$0.13 \pm 0.03$	$0.23 \pm 0.02$

Intra-observer variability has been evaluated by comparing the two sets of manually drawn contours (table 1). Auto-contouring is shown to have performed well, with conformity metrics almost as good as the intra-observer values: the CI has median $0.820 \pm 0.002$ and mean $0.810 \pm 0.002$, the DBC has median ($0.64 \pm 0.01$) mm and mean ($0.69 \pm 0.01$) mm, the mean DTC is ($0.03 \pm 0.01$) mm, the mean difference in left–right dimensions is ($-0.25 \pm 0.02$), and the mean difference in anterior-posterior dimensions is ($0.23 \pm 0.02$) mm.

### 3.3. Conformity analysis: 30 patients

To check the robustness of the auto-contouring technique against a broader range of patient anatomies, the performance has been analysed for the 30 patients for whom the spinal cord has been manually outlined once on a single randomly selected MVCT scan per patient. This dataset consists of 30 MVCT scans and 663 slices. Results obtained (table 2) are comparable with those from the 3-patient study.

**Table 2.** Results from outlining the spinal cord on one randomly chosen MVCT scan for each of 30 patients, comparing auto-contours and manually drawn contours. The numbers of MVCT scans and slices are indicated. Results are given for the median, mean and standard deviation of the conformity index, and of the distance between centres (mm); and for the mean of the distance (mm) to conformity, of the difference (mm) in left–right dimensions, and of the difference (mm) in anterior-posterior dimensions. Quoted uncertainties are statistical.

Patients	30
Scans	30
Slices	663
Median CI	$0.740 \pm 0.005$
Mean CI	$0.725 \pm 0.004$
Std Dev CI	0.105
Median DBC	$1.01 \pm 0.04$
Mean DBC	$1.17 \pm 0.03$
Std Dev DBC	0.73
Mean DTC	$0.16 \pm 0.02$
Mean L-R diff	$0.59 \pm 0.06$
Mean A-P diff	$0.14 \pm 0.07$

### 3.4. Conformity analysis: inter-observer study

**Figure 4.**
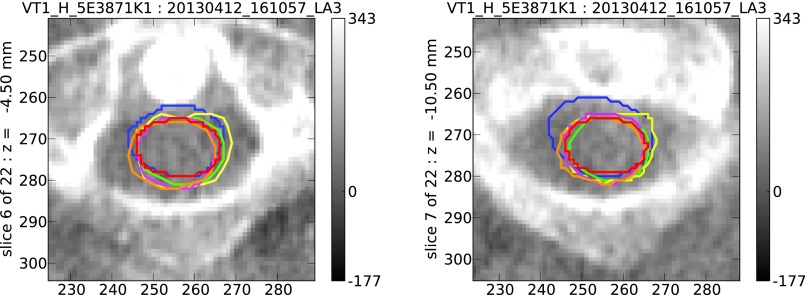
Two example MVCT scan slices, at different levels. Outlines of the spinal cord have been drawn manually, and independently, by six oncologists, each represented by a different colour.

To gain further insight into the accuracy of the auto-contours, each of six oncologists has independently outlined the spinal cord (figure 4) on the same six MVCT scans. These included two MVCT scans, chosen at random, from each of the initial three patients. The contours drawn by the six oncologists are generally in reasonable agreement, and all would be clinically acceptable, but some variability is evident. A conformity analysis has been carried out, where the contours from different oncologists have been compared with one another (table 3), and with the corresponding auto-contours (table 4).

**Table 3.** Inter-observer variability, from comparisons between contours of the spinal cord drawn by each of six oncologists and the contours drawn by the other five. The numbers of MVCT scans and slices relative to each comparison are shown. Results are given for the median and mean of the conformity index, and of the distance between centres (mm); and for the mean of the difference (mm) in left–right dimensions, and of the difference (mm) in anterior-posterior dimensions. Quoted uncertainties are statistical.

Observer	1	2	3
Patients	3	3	3
Scans	6	6	6
Slices	133	133	133
Median CI	$0.723 \pm 0.007$	$0.763 \pm 0.005$	$0.753 \pm 0.005$
Mean CI	$0.713 \pm 0.005$	$0.756 \pm 0.004$	$0.742 \pm 0.004$
Median DBC	$1.02 \pm 0.05$	$0.82 \pm 0.03$	$0.88 \pm 0.03$
Mean DBC	$1.06 \pm 0.04$	$0.89 \pm 0.03$	$0.90 \pm 0.02$
Mean L-R diff	$-0.1 \pm 0.1$	$0.2 \pm 0.1$	$-1.4 \pm 0.1$
Mean A-P diff	$-1.4 \pm 0.1$	$-0.3 \pm 0.1$	$-0.2 \pm 0.1$

Observer	4	5	6
Patients	3	3	3
Scans	6	6	6
Slices	133	133	133
Median CI	$0.764 \pm 0.005$	$0.745 \pm 0.006$	$0.651 \pm 0.009$
Mean CI	$0.756 \pm 0.004$	$0.739 \pm 0.004$	$0.643 \pm 0.007$
Median DBC	$0.91 \pm 0.03$	$0.90 \pm 0.04$	$1.24 \pm 0.06$
Mean DBC	$0.94 \pm 0.02$	$0.96 \pm 0.03$	$1.32 \pm 0.05$
Mean L-R diff	$-0.1 \pm 0.1$	$-0.4 \pm 0.1$	$1.7 \pm 0.2$
Mean A-P diff	$-0.2 \pm 0.1$	$-0.1 \pm 0.1$	$2.2 \pm 0.1$

**Table 4.** Comparisons for six MVCT scans between auto-contours of the spinal cord and contours drawn by each of six oncologists. Results are given for the median and mean of the conformity index, and of the distance between centres (mm). Quoted uncertainties are statistical.

Observer	1	2	3
Median CI	$0.78 \pm 0.01$	$0.79 \pm 0.01$	$0.77 \pm 0.01$
Mean CI	$0.77 \pm 0.01$	$0.78 \pm 0.01$	$0.75 \pm 0.01$
Median DBC	$0.70 \pm 0.05$	$0.67 \pm 0.04$	$0.74 \pm 0.06$
Mean DBC	$0.77 \pm 0.04$	$0.77 \pm 0.04$	$0.88 \pm 0.05$

Observer	4	5	6
Median CI	$0.76 \pm 0.01$	$0.74 \pm 0.01$	$0.64 \pm 0.01$
Mean CI	$0.75 \pm 0.01$	$0.73 \pm 0.01$	$0.62 \pm 0.01$
Median DBC	$0.92 \pm 0.06$	$1.03 \pm 0.07$	$1.37 \pm 0.09$
Mean DBC	$1.02 \pm 0.05$	$1.11 \pm 0.05$	$1.54 \pm 0.07$

The oncologist identified as observer 6 may be regarded as an outlier. The contours drawn by this oncologist are systematically a little larger than those of the others: their mean left–right dimension is greater by ($1.7 \pm 0.2$) mm, and their mean anterior-posterior dimension is greater by ($2.2 \pm 0.1$) mm. Each of observers 1 to 5 has a mean conformity index relative to the other five in the range $0.713 \pm 0.005$ to $0.756 \pm 0.004$, whereas the value for observer 6 is $0.643 \pm 0.007$.

The contours of the spinal cord on the kVCT scans that were propagated to give the auto-contours on the MVCT scans were drawn by the oncologist identified as observer 1. It might be suspected that this could bias the auto-contours to agree better with the contours drawn manually on the MVCT scans by observer 1. There is little evidence that this is the case. The mean conformity index is in the range $0.73 \pm 0.01$ to $0.78 \pm 0.01$ for observers 2 to 5, and is $0.77 \pm 0.01$ for observer 1. The level of agreement between auto-contours and contours drawn manually is comparable with the inter-observer variability of the six oncologists.

### 3.5. Delivered dose to spinal cord

**Figure 5.**
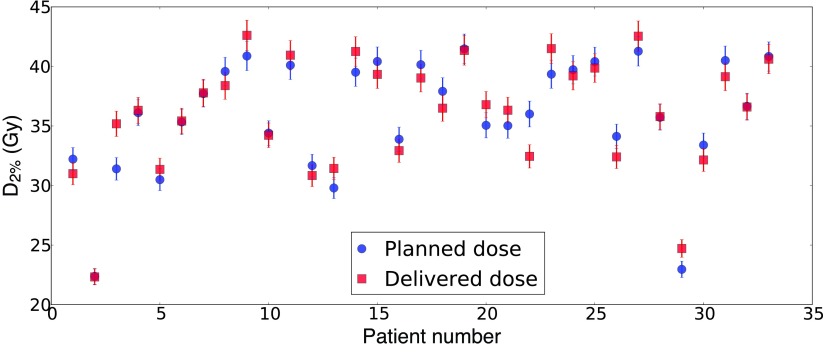
Near-maximum dose, $D_{2\%}$, to the spinal cord, as planned (blue circles) and as delivered (red squares), for 33 patients who underwent radiotherapy for head-and-neck cancers.

The auto-contours generated are sufficiently robust to permit calculation of delivered dose. Since the spinal cord is a serial organ (International Commission on Radiation Units and Measurements 1999), the dose parameter considered is the near-maximum dose $D_{2\%}$ (International Commission on Radiation Units and Measurements 2010), defined as the minimum dose to the 2% of the organ volume where dose is highest. An analysis carried out for the 33 patients considered in our conformity studies indicates that planned and delivered values of $D_{2\%}$ agree within their statistical uncertainties (figure 5).

We did not accumulate delivered dose using rigid registration only, as deformable registration improved automated contouring performance. The greater variability in anterior/posterior curvature of the inferior neck also means that deformable registration may be more important for accurate accumulation in this region, although doses in this region are generally lower.

The delivered value of $D_{2\%}$ was higher than planned in 15 cases, with a mean excess of 1.3 Gy, and was lower than planned in 18 cases, with a mean deficit of 1.0 Gy. Results suggest no systematic tendency for the delivered $D_{2\%}$ value to be larger or smaller than planned. In the most extreme case (patient numbered 3), the planned $D_{2\%}$ value of 31.4 Gy was exceeded by 3.8 Gy. Even here, the higher delivered dose was not at a level that would have raised the risk of late damage to the spinal cord. However, if the planned dose were on the limit of tolerance, the recorded increase might be important clinically.

The technique developed for computing delivered dose has potential for automated dose monitoring over a course of treatment. Treatment could be replanned for patients receiving a higher dose than expected to the spinal cord, which could be at risk of damage. Treatment could also be replanned for patients receiving a lower dose than expected to the spinal cord, where there could be benefits in escalating the dose to the tumour, or in reducing doses to other organs at risk.

## 4. Conclusions

Current commerical solutions offer segmentation of the spinal canal, rather than of the cord. We have developed a method for automated segmentation of the spinal cord on MVCT image-guidance scans, using image-registration transforms to propagate contours drawn manually on kVCT planning scans. An affine transform followed by a B-spline transform, with control-point spacing of 15 mm, allows conformity that parallels human expert observers. Resulting auto-contours agree well with contours drawn manually on the MVCT scans. When considering all 30 MVCT scans for each of three patients (2107 scan slices), the median conformity index is $0.759 \pm 0.003$, and the median distance between centres is ($0.87 \pm 0.01$) mm. When considering a single MVCT scan for each of 30 patients (663 scan slices), the median conformity index is $0.740 \pm 0.005$, and the median distance between centres is ($1.01 \pm 0.04$) mm. The values of the conformity metrics compare favourably with those that we find for intra-observer and inter-observer variability.

Using auto-contours, we have computed the delivered dose to the spinal cord for a set of 33 patients, demonstrating the potential for dose monitoring over a course of treatment. The methodology that we have developed is being used in the VoxTox study to compute delivered dose to the spinal cord for a cohort of several hundred patients, allowing investigation of correlations between delivered dose and toxicity.
